# Training and Technical Assistance for Compliance With Beverage and Physical Activity Components of New York City’s Regulations for Early Child Care Centers

**DOI:** 10.5888/pcd11.130434

**Published:** 2014-10-16

**Authors:** Jakub Kakietek, Lillian Dunn, Sarah Abood O’Dell, Jan Jernigan, Laura Kettel Khan

**Affiliations:** Author Affiliations: Lillian Dunn, New York City Department of Health and Mental Hygiene, New York, New York; Sarah Abood O’Dell, ICF International, Atlanta, Georgia; Jan Jernigan, Laura Kettel Khan, Centers for Disease Control and Prevention, Atlanta, Georgia.

## Abstract

**Introduction:**

In 2006, the New York City Department of Health and Mental Hygiene (DOHMH) passed regulations for child care centers that established standards for beverages provided to children and set a minimum amount of time for daily physical activity. DOHMH offered several types of training and technical assistance to support compliance with the regulations. This article analyzes the association between training and technical assistance provided and compliance with the regulations in a sample of 174 group child care centers.

**Methods:**

Compliance was measured by using a site inventory of beverages stored on premises and a survey of centers’ teachers regarding the amount of physical activity provided. Training and technical assistance measures were based on the DOHMH records of training and technical assistance provided to the centers in the sample and on a survey of center directors. Ordinal logistic regression was used to assess the association between training and technical assistance measures and compliance with the regulations.

**Results:**

Measures of training related to physical activity the center received: the number of staff members who participated in Sport, Play and Active Recreation for Kids (SPARK) and other training programs in which a center participated were associated with better compliance with the physical activity regulations. Neither training nor technical assistance were associated with compliance with the regulations related to beverages.

**Conclusion:**

Increased compliance with regulations pertaining to physical activity was not related to compliance with beverage regulations. Future trainings should be targeted to the specific regulation requirements to increase compliance.

## Introduction

The obesity epidemic among children is a substantial public health concern in the United States (1). Environment and policy change interventions in child care settings are a promising way of responding to this epidemic (2–4). Policy interventions for obesity prevention often target children in settings such as schools and early child care and education centers, places where children spend large amounts of time (5,6). State and local health departments, identified as key partners in supporting community-based obesity prevention, often provide training and technical assistance to improve centers’ ability to comply with such regulations (7). The emerging consensus that policy changes are an important public health tool for addressing childhood obesity makes it necessary to examine the factors that facilitate the implementation of and compliance with policy-based interventions. This article analyzes the association between the New York City Department of Health and Mental Hygiene (DOHMH) regulations governing beverages and physical activity in group child care centers and training programs and technical assistance offered by the DOHMH to support and increase compliance with the regulations.

The New York City regulations, adapted in 2006, set standards for beverages served and strengthen requirements for physical activity offered. Child care centers are required to serve only milk with 1% or less fat to children aged 2 years or older; provide only 100% fruit juice in servings of no more than 6 ounces per day; make water available and accessible throughout the day, including at meals; and they are prohibited from serving beverages with added sweeteners. Child care centers are also required to provide at least 60 minutes of physical activity a day. At least 30 minutes of the total physical activity provided must be structured (ie, teacher-led).

To support adherence to these regulations and to encourage healthy habits in early childhood, the DOHMH provided nutrition- and physical activity-related training programs and technical assistance to licensed group child care centers. The training programs — including Sport, Play and Active Recreation for Kids (SPARK), Eat Well, Play Hard (EWPH), and the EWPH Training of Teachers (TOTs) — were designed and implemented to ensure that all child care centers were given the resources and guidance necessary to improve staff knowledge related to nutrition and classroom physical activity and help increase compliance with the regulations. SPARK training sessions reviewed and discussed the new beverage, physical activity, and screen time regulations in addition to the physical activity curriculum; EWPH and TOTs did not.

The key research hypothesis tested here is that training and technical assistance are associated with better compliance. Analyses presented are part of the larger multi-method evaluation that also examines the impact of compliance on child-level outcomes such as physical activity and beverage consumption (8,9).

## Methods

### Participants

This cross-sectional study focused on the 1,654 early child care and education centers licensed by the New York City DOHMH Bureau of Child Care. To support child care centers in underserved communities, DOHMH maintains Department of Public Health Offices (DPHOs) that provide technical assistance and other services to child care centers in DPHO catchment areas. Although nearly all (301 of 311) of the child care centers in DPHO catchment areas were in areas with high levels of poverty (census tracts with 40% or more of families with incomes at 200% of the federal poverty line or below), only about 41% (549 of 1,343) of the non-DPHO centers were in neighborhoods with high poverty levels. To ensure comparability between DPHO and non-DPHO centers, only centers in low income, non-DPHO areas were included in the sampling frame. The final sampling frame included 301 of the 311 child care centers in DPHO neighborhoods and 350 child care centers in 9 non-DPHO neighborhoods. Of these, 260 centers were randomly sampled (130 in DPHO neighborhoods and 130 in non-DPHO neighborhoods). Ten percent (26) of the centers were ineligible for the study because they had an insufficient number of children (fewer than 10), had no children in the target age group (3 or 4 years), enrolled only special needs children, were closing or had already closed, or were unreachable. Of the 234 eligible centers, 58 (25%) refused to participate. At the end of the sample selection, data was collected in 176 centers. Complete data was available for 174 centers (92 in a DHPO area and 82 outside) (Figure).

**Figure:**
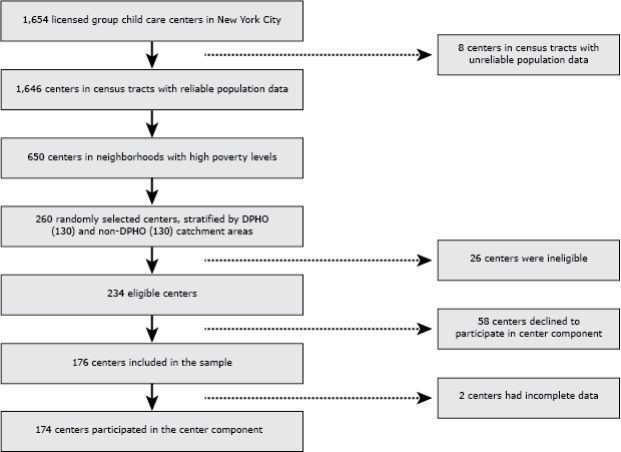
Sample Flow of Participants in New York City Child Care Centers (n = 174), 2010.

### Training and technical assistance offered by DOHMH

The DOHMH offered full-day training sessions for child care center staff on a modified SPARK Early Childhood curriculum to ensure that center staff had the skills to provide 30 minutes of structured physical activity daily. Participants learned how to lead students through structured activities for small classroom spaces and received a manual and equipment necessary for physical activity lessons demonstrated in the training. In response to demand from trained child care center staff, the DOHMH offered a second full-day SPARK training for staff who had participated in the first training.

Centers located in the DPHO catchment areas that target low-income neighborhoods were all provided additional on-site training that focused on that center’s specific issues with compliance related to nutrition and physical activity in general and specifically to the new regulations. This individualized technical assistance ended after all centers had been visited on at least 2 occasions regardless of their compliance.

The DOHMH also offered the EWPH program to provide information about healthy eating habits to children, staff, and parents. Unlike SPARK, EWPH was not designed to assist centers in complying with the regulations and did not address the regulations specifically; instead, EWPH reinforced concepts related to nutrition and physical activity that complemented the regulations. Centers participating in EWPH received 8 classroom lessons led by dietitians that focused on the importance of nutrition, portion size, and family meals; lessons on role modeling and healthy eating for staff; and lessons for parents on how to make nutritious and inexpensive meals at home. Centers that participated in EWPH workshops were eligible to participate in the TOTs program, which trained staff members to implement the EWPH nutrition curriculum at their center.

Centers included in the evaluation also reported participation in other training programs not offered by the DOHMH. These trainings included, but were not limited to, Administration for Children’s Services (ACS) and Child and Adult Care Feeding Program (CACFP) workshops on nutrition and physical activity, I am Moving, I am Learning, Go! Healthy, and other programs.

### Measures

Data collection was conducted by using site inventories and in-person interviews with child care center directors, teachers, and food service staff. The site inventory included items related to availability of and access to play space, availability of water, and types of beverages served, including milk and juice. The interviews collected information on the amount of physical activity provided to children, and other center characteristics. Survey items were adapted from existing validated instruments designed for similar populations (10). Instruments are available on request. Data on training (including SPARK, EWPH, and TOTs) and technical assistance the centers had received were obtained from DOHMH records.

### Independent variables: training and technical assistance

Data on a center’s participation in training was captured through director interviews and the DOHMH’s records. Directors were asked whether their center had participated in SPARK and EWPH and whether the director had attended a SPARK training. The DOHMH provided information on the number of staff members from each center who participated in the EWPH or TOTs trainings and the 2 SPARK training sessions. The DOHMH provided the number of staff from each center who participated in the first and second SPARK training sessions during the 12 months before the evaluation. For each center, those numbers were used as the measure of staff participating in SPARK training.

Center directors were also asked about participation in other nutrition and physical activity training programs. Because supplemental technical assistance was provided by the DOHMH to all centers within the 3 DPHO catchment areas, center location served as an indicator for the additional technical assistance provided by the department in the regression models.

### Dependent variables: assessing compliance

Measures of compliance with the regulations related to juice, milk, and sugar-sweetened beverages (SSBs) were based on data collected through the site inventory, while compliance with the regulations related to the availability of water and physical activity were based on self-report of center staff. For each specific beverage regulation, centers were considered compliant if the site inventory found only milk with 1% or less fat, only 100% fruit juice, and no beverages with added sweeteners. For regulations concerning water, centers were considered compliant if center staff reported that water was available to children throughout the day. For each physical activity regulation, centers were considered compliant if center staff reported that children received at least 30 total minutes of structured physical activity per day and at least 60 minutes of total physical activity. Two additive scores were constructed, one for compliance with beverage regulations and another for compliance with physical activity. The beverage score ranged from 0 (did not comply with any of the 4 beverage regulation components) to 4 (complied with all 4 beverage-related regulations components). The physical activity score ranged from 0 (did not comply with either of the 2 components on physical activity) to 2 (complied with both components on physical activity).

### Analysis

Multivariate ordinal logistic regression models were used to examine the association between compliance and training and technical assistance. Control variables included in the models captured aspects of center size: average classroom size (average number of students per classroom) and student–teacher ratio; infrastructure: presence or absence of indoor and outdoor play spaces (captured through the site inventory); staffing: presence of dedicated food staff and teaching staff turnover; participation in federal programs related to nutrition or physical activity: CACFP and Head Start; and proxy measures of the director’s leadership: director’s tenure (number of years at the center) and educational attainment. These variables were significantly associated with compliance in bivariate analysis. Variables related to nutrition (eg, presence of dedicated food service staff, participation in EWPH) were included only in the model of beverage compliance; variables pertaining to physical activity (eg, presence of outdoor physical activity facilities, participation in SPARK) were included only in the model of physical activity compliance.

Data on compliance with the 100% juice regulation were not available for one center and data on compliance with the SSB regulation were not available for 2 centers. The final sample used in the multivariate models included 174 of the 176 centers. All analyses were conducted using STATA version 9 (StataCorp).

## Results

We found that 92 centers (52.9%) were located in DPHO technical assistance areas (Table 1). A total of 151 centers (86.8%) participated in SPARK; directors in 93 centers (46.5%) were trained as part of the program. In an average center, 9 teachers participated in the first SPARK training and 1 teacher participated in the follow-up (second) training. A total of 38 (21.8%) centers participated in one training related to physical activity other than SPARK, and 3 centers (1.7%) participated in 2 such trainings. A total of 53 centers (30.5%) participated in EWPH. On average, 0.5 teachers per center participated in EWPH TOT. A total of 93 (53.4%) centers participated in one training related to nutrition other than EWPH, and 15 centers (8.6%) participated in 2 such programs. A total of 98 centers (56.3%) participated in Head Start and 48 (27.6%) in CACFP. A total of 105 (60.3%) center directors served in that position for more than 5 years and 147 (84.5%) had a graduate degree. A total of 154 centers (88.5%) had dedicated food service staff, 61 (35.1%) had indoor physical activity facilities, 126 (72.4%) had private outdoor facilities for physical activity, and 30 (17.25) had access to shared outdoor facilities (eg, park) for physical activity. An average center was open for 10 hours during the day, had about 6 students per teacher, and had a teaching staff turnover ratio of 0.1.

**Table 1:** Training and Technical Assistance to Improve Nutrition and Physical Activity in 174 New York City Child Care Centers, 2010

Categorical Variables	N (%)
**Center located in the DPHO area^a^**	
Yes	92 (52.9)
No	82 (47.1)
**Center participated in SPARK^b^**	
Yes	151 (86.8)
No	23 (13.2)
**Center participated in EWPH^c^**	
Yes	53 (30.5)
No	121 (69.5)
**Director reported participation in SPARK^d^**	
Yes	93 (46.5)
No	81 (53.5)
**No. of physical activity–related trainings other than SPARK and EWPH in which the center participated**	
0	133 (76.4)
1	38 (21.8)
2	3 (1.7)
**No. of nutrition-related trainings other than SPARK^b^ and EWPH^c^ in which the center participated**	
0	66 (37.9)
1	93 (53.4)
2	15 (8.6)
**Continuous variables, m**e**an (SD)**	
No. of teachers trained in the 1st SPARK^b^ workshop	8.6 (9.0)
No. of teachers trained in the 2nd SPARK^b^ workshop	1.2 (3.6)
No. of teachers who participated in TOTs^e^	0.5 (2.4)
**Continuous variables, n (%)**	
**Center participates in Head Start^f^**	
Yes	98 (56.3)
No	76 (43.7)
**Center participates in CACFP^g^**	
Yes	48 (27.6)
No	126 (72.4)
**Center is part of a larger agency**	
Yes	69 (39.7)
No	105 (60.3)
**Director’s tenure (years at the center)**	
1–3 years	46 (26.5)
3–5 years	23 (13.2)
More than 5 years	105 (60.3)
**Director’s educational attainment**	
No bachelor’s degree	8 (4.6)
Bachelor’s degree	19 (10.9)
Graduate degree	147 (84.5)
**Center has dedicated food service staff^h^**	
Yes	154 (88.5)
No	20 (11.5)
**Center has indoor physical activity facilities^h^**	
Yes	61 (35.1)
No	113 (64.9)
**Center has private outdoor physical activity facilities^h^**	
Yes	126 (72.4)
No	48 (27.6)
**Center has shared outdoor physical activity facilities^h^**	
Yes	30 (17.2)
No	144 (82.8)
**Continuous variables, mean (SD)**	
Average classroom size (children aged 3–4 y)	6.7 (3.1)
No. of hours of service	10 (1.2)
Student-teacher ratio	5.7 (3.0)
Teaching staff turnover ratio^i^	0.1 (0.2)

a DPHO (District Public Health Offices) is a program of the New York City DOHMH that targets resources to high-need neighborhoods in the South Bronx, East and Central Harlem, and North and Central Brooklyn. These centers all received 2 individualized on-site technical assistance sessions.

b SPARK (Sport, Play and Active Recreation for Kids) is a physical activity training program that New York City Department of Health and Mental Hygiene (DOHMH) provides free of charge to licensed child care centers.

c EWPH (Eat Well Play Hard) is a childhood obesity-prevention initiative of the New York State Department of Health. The EWPH intervention is a 6-week training program provided free of charge by DOHMH to child care centers where at least 50% of the enrolled students are eligible for free or reduced-price meals.

d Coded 1 if the director reported she or he participated in the SPARK training and 0 otherwise.

e TOTS (Training of Teachers) is a DOHMH technical assistance program that provides child care center staff the skills necessary to lead the EWPH nutrition and physical activity curriculum in their classrooms.

f Head Start is a comprehensive developmental program for preschool-aged children and their families who earn a household income below the federal income poverty threshold and is administered by the Administration for Children and Families, US Department of Health and Human Services.

g CACFP (Child and Adult Care Food Program) is administered by the US Department of Agriculture through federal grants to state health departments to provide nutritious meals and snacks to low-income individuals.

h Presence or absence of variables coded 1 if the appropriate staff or facilities are present and 0 otherwise.

i Number of new staff hired during the 12 months preceding the study divided by the total number of staff.

We calculated the number and percentage of centers in the sample that reported compliance with individual regulation components (Table 2). Compliance with components of regulations on beverages and physical activity ranged from 63.4% to 86.4%.

**Table 2:** Compliance With Beverage and Physical Activity Regulations, New York City Child Care Centers (n = 174), 2010

Regulation	Centers That Comply, n (%)
**Beverages served**	
Milk that is served has a 1% fat content or less	133 (75.6)
Only 100% juice is served	111 (63.4)
Beverages with added sweeteners are not provided	142 (81.6)
Water is readily available to children throughout the day, including meal times	152 (86.4)
**Beverage compliance score^a^ (range: 0–4)**	
Noncompliance, 0	1 (0.6)
1	12 (6.9)
2	30 (17.3)
3	57 (32.9)
Total compliance, 4	73 (42.3)
**Physical activity offered**	
Children are offered at least 30 min of structured physical activity a day	134 (77.5)
Children are offered at least 60 min of physical activity a day	148 (85.5)
**Physical activity compliance score^b^ (range: 0–2)**	
Noncompliance, 0	20 (11.6)
Compliance with 1 regulation, 1	24 (13.9)
Total compliance, 2	129 (74.5)

a Beverage score ranged from 0 (centers that served milk with more than 1% fat, provided juice drinks that were not 100% fruit juice, provided sugar-sweetened beverages, and did not make water readily available) to 4 (centers that served only milk with 1% or less fat, 100% fruit juice, did not provide sugar-sweetened beverages, and made water readily available).

b Physical activity score ranged from 0 (centers that reported offering fewer than 30 min of structured physical activity and fewer than 60 min of total physical activity a day) to 2 (centers that reported offering children 30 or more minutes of structured physical activity and 60 or more minutes of total physical activity a day).

Results of the regression models for beverages and physical activity (Table 3) show that no training or technical assistance indicators were associated with compliance. Participation in CACFP and center’s operating hours were significantly associated with compliance. Centers that participated in CACFP had 3.5 times higher odds of compliance with an additional beverage-related regulation than centers that did not participate in the CACFP program (AOR 3.47, 95% confidence interval [CI], 1.39–8.66). Each additional hour a center was open was associated with a 28% decrease in the odds of being in compliance with an additional beverage regulation (AOR 0.72, 95% CI, 0.54–0.97).

**Table 3:** Association Between Compliance with Beverage and Physical Activity Regulations and Training and Technical Assistance Based on Estimates of Ordinal Logistical Regression Models, New York City Child Care Centers (n = 174), 2010

Center Characteristics	Model 1	Model 2
	Beverage Compliance Score	Physical Activity Compliance Score
	AOR (95% CI)	AOR (95% CI)
Center participates in Head Start^a^	1.49 (0.67–3.34)	0.35 (0.12–1.02)
Center participates in CACFP^b^	3.47 (1.39–8.66)	0.93 (0.30–2.90)
Center is part of a larger agency	0.81 (0.43–1.53)	0.68 (0.30–1.57)
Average classroom size^c^	1.03 (0.95–1.13)	0.87 (0.76–0.98)
No. of hours the center is opened during the day	0.72 (0.54–0.97)	0.60 (0.39–0.92)
Student–teacher ratio: 5.7^d^	0.91 (0.82–1.01)	1.05 (0.90–1.21)
Teaching staff turnover ratio: 0.1^e^	0.35 (0.10–1.27)	0.11 (0.02–0.53)
**Director’s tenure (number of years at the center)**		
3–5 years	1.03 (0.34–3.08)	0.64 (0.16–2.56)
More than 5 years	0.51 (0.25–1.02)	1.21 (0.48–3.05)
**Director’s educational attainment**		
No bachelor's degree	0.45 (0.11–1.88)	0.68 (0.12–3.82)
Bachelor’s degree	0.60 (0.24–1.53)	0.74 (0.19–2.81)
Center has dedicated food service staff^f^	1.34 (0.46–3.89)	—
Center has indoor physical activity facilities^f^	—	0.69 (0.28–1.69)
Center has private outdoor physical activity facilities^f^	—	3.67 (1.47–9.13)
Center has shared outdoor physical activity facilities^f^	—	1.04 (0.33–3.27)
Center is in the DPHO area^g^/DPHO technical assistance	0.79 (0.39–1.61)	1.33 (0.50–3.50)
Center participated in SPARK^h^	—	0.71 (0.21–2.44)
Center participated in EWPH^i^	1.33 (0.58–3.03)	0.45 (0.15–1.36)
Director reported participation in SPARK^j^	—	2.27 (0.96–5.37)
No. of physical activity–related trainings other than SPARK^h^ and EWPH^i^ in which the center participated	—	3.57 (1.28–10.01)
No. of nutrition-related trainings other than SPARK^h^ and EWPH^i^ in which the center participated	1.43 (0.86–2.37)	—
No. of teachers trained in the first SPARK^h^ workshop	—	1.09 (1.01–1.17)
No. of teachers trained in the 2nd SPARK^h^workshop	—	1.13 (0.82–1.55)
No. of teachers who participated in TOTs^k^	1.23 (0.94–1.63)	1.07 (0.86–1.33)
*P* value (χ^2^)	<.001	.004
Pseudo R^2^	0.124	0.169

Abbreviations: AOR, adjusted odds ratio; CI, confidence interval; —, not applicable; CACFP, Child and Adult Care Food Program; DPHO, Department of Public Health Office; EWPH, Eat Well Play Hard; SPARK, Sport, Play and Active Recreation for Kids; TOTs, Training of Teachers.

a Head Start is a comprehensive developmental program for preschool-aged children and their families who earn household income below the federal income poverty threshold administered by the Administration for Children and Families within the US Department of Health and Human Services.

b CACFP is a program of the US Department of Agriculture that administers federal grants to state health departments to provide nutritious meals and snacks to low-income individuals.

c Average number of students per classroom.

d Number of students in the center divided by the no. of teachers in the center.

e Number of new staff hired during the 12 months preceding the study divided by the total no. of staff.

f Presence or absence variables: coded 1 if the appropriate staff or facilities were present and 0 otherwise.

g DPHO is a program of the New York City DOHMH that targets resources to high need neighborhoods in the South Bronx, East and Central Harlem, and North and Central Brooklyn. These centers received 2 individualized on-site technical assistance sessions.

h SPARK is a physical activity training program New York City DOHMH provides free of charge to licensed child care centers.

i EWPH is a childhood obesity initiative of the New York State Department of Health. EWPH intervention involves a 6-week training program provided free of charge by the New York City Department of Health and Mental Hygiene to child care centers where at least 50% of the enrolled students are eligible for free or reduced-price meals.

j Coded 1 if the director reported she or he participated in the SPARK training and 0 otherwise.

k TOTS is a New York City DOHMH technical assistance program that provides child care center staff the skills necessary to lead the EWPH nutrition and physical activity curriculum in their classrooms.

In the physical activity model (Table 3), 2 indicators of physical activity training, but not technical assistance, were associated with compliance: 1) the number of teachers who participated in the first SPARK training and 2) the number of physical activity training programs other than SPARK in which a center participated. Each additional teacher who participated in the first SPARK training was associated with an increase of about 9% in the odds of compliance with an additional regulation (AOR 1.09; 95% CI, 1.01–1.17). Each additional physical activity training program other than SPARK was associated with a 3.6 times increase in the odds of compliance with an additional regulation pertaining to physical activity (AOR 3.57; 95% CI, 1.28–10.01).

In the physical activity model, an increase of one child in the average classroom size for children aged 3 or 4 was associated with a decrease of about 13% in the odds that the center would comply with an additional physical activity regulation (AOR, 0.87; 95% CI, 0.76–0.98). Also, each additional hour the center reported being open was associated with a percent decrease of about 41% in the odds that the center would comply with an additional regulation (AOR, 0.60; 95% CI, 0.39–0.92). An increase of 1 in the teaching staff turnover ratio was associated with a 90% decrease in the odds that the center would comply with an additional regulation (AOR, 0.11; 95% CI, 0.02–0.53). Centers that had their own outdoor facilities for physical activity had almost 3.6 times higher odds of compliance with an additional physical activity regulation than centers that did not have such facilities (AOR 3.67, 95% CI 1.47–9.13).

## Discussion

Few empirical studies have systematically assessed compliance with regulations concerning nutrition and physical activity in child care settings. Recent assessments of compliance with nutrition and physical activity regulations among child care centers in Delaware found that 86% of child care centers were compliant with state-wide recommendations (not regulations) regarding unstructured physical activity and 78% compliant with the recommendations concerning structured physical activity (11). These findings are consistent with ours: 77.5% of centers were compliant with the regulations regarding structured physical activity and 85.5% of centers were compliant with the regulation concerning total physical activity. An assessment of compliance with regulations related to nutrition conducted in Delaware found that 88.3% of the centers were compliant with state regulations concerning the types of juice served (12). Our study showed that only 63% of centers were compliant with the regulations concerning juice. This difference may reflect actual differences in compliance, or it may be that the Delaware compliance measure (which was based on self-report) was inflated because of social desirability bias. Observed compliance with regulations concerning water (86.4%) is consistent with other evaluations conducted in Delaware (12) and Connecticut (13): 82.1% and 84%, respectively.

We are not aware of any studies that examined directly the effects of training and technical assistance to improve compliance with regulations concerning nutrition and physical activity in child care settings in the United States. One recent study examined the effect of training on child care staff’s knowledge of regulations concerning nutrition, physical activity, and screen time (14). However, it did not address the extent to which the training was associated with improved compliance. Our evaluation supports the potential effect of training and technical assistance programs on a center’s compliance with the New York City regulations. Both the number of staff who participated in SPARK and the number of training programs related to physical activity other than SPARK in which a center participated were significantly and positively associated with physical activity regulation compliance. Overall, the results suggest that training sessions such as those offered by the DOHMH may offer child care center staff concrete tools and methods to improve the implementation of physical activity regulations and thereby improve physical activity compliance, but training and technical assistance is less important for beverage regulation compliance.

The lack of association between the second SPARK training and compliance with the regulations suggests that the first training, which provided teachers with basic skills to lead physical activity sessions in their centers, was sufficient to support compliance.

The difference in the associations for physical activity and beverage-related training and technical assistance may have resulted from different ways that physical activity and beverages offered at a center can be influenced and improved through training programs. Simple knowledge of what beverages should be served is insufficient to improve compliance. Some researchers suggest that high prices of healthy foods and beverages may present a barrier to providing them in health care settings (15).

Our analysis showed that factors such as classroom size, operating hours, and teaching staff turnover were associated with regulatory compliance. Consistent with the previous findings, our analysis showed that participation in CACFP was associated with better compliance with regulations related to nutrition (12) and that compliance with regulations related to physical activity was associated with the presence of physical activity facilities at the center (11).

One limitation of this evaluation is that it is based on a cross-sectional design and cannot accurately delineate the direction of the causal linkages between compliance and training and technical assistance. Centers that were dedicated to high standards in nutrition and physical activity and complied with the regulations may have been more likely to participate in training and technical assistance programs than less dedicated and compliant centers. Another limitation is that measures of compliance with regulations on physical activity and water were based on self-report, which might have introduced social desirability bias and may have inflated our estimates. We minimized the effects of measurement bias due to self-report by using, where possible, measures based on site inventories and observational data. The evaluation was also limited by being conducted in group child care centers in low-income communities in New York City. Although the study’s setting may limit generalizability, it does give some indication as to how urban child care centers in resource-poor communities may respond to regulations related to nutrition and physical activity.

Our findings have important implications for other jurisdictions considering similar regulations. First, they suggest that training programs can increase compliance with regulations pertaining to physical activity but not to beverages. Therefore, jurisdictions adopting new policies may consider providing training focused on physical activity. Second, numerous center characteristics such as large classroom size, high teaching staff turnover, and center open for long hours are negatively associated with compliance. Training to improve compliance may help offset the effect of those characteristics. Consequently, local health departments may want to focus their training on large centers with high staff turnover (including repeating training for new staff), large classroom size, and centers with long hours of service. Finally, because training appears to have less influence on compliance for beverages, tools such as a simple checklist of which beverages are and are not acceptable may be adequate.
